# Actionable mutations in canine hemangiosarcoma

**DOI:** 10.1371/journal.pone.0188667

**Published:** 2017-11-30

**Authors:** Guannan Wang, Ming Wu, Martha A. Maloneyhuss, John Wojcik, Amy C. Durham, Nicola J. Mason, David B. Roth

**Affiliations:** 1 Department of Pathology and Laboratory Medicine, Raymond and Ruth Perelman School of Medicine, University of Pennsylvania, Philadelphia, PA, United States of America; 2 Illumina, San Diego, CA, United States of America; 3 Department of Clinical Studies, School of Veterinary Medicine, University of Pennsylvania, Philadelphia, PA, United States of America; 4 Department of Pathobiology, School of Veterinary Medicine, University of Pennsylvania, Philadelphia, PA, United States of America; Ohio State University Wexner Medical Center, UNITED STATES

## Abstract

**Background:**

Angiosarcomas (AS) are rare in humans, but they are a deadly subtype of soft tissue sarcoma. Discovery sequencing in AS, especially the visceral form, is hampered by the rarity of cases. Most diagnostic material exists as archival formalin fixed, paraffin embedded tissue which serves as a poor source of high quality DNA for genome-wide sequencing. We approached this problem through comparative genomics. We hypothesized that exome sequencing a histologically similar tumor, hemangiosarcoma (HSA), that occurs in approximately 50,000 dogs per year, may lead to the identification of potential oncogenic drivers and druggable targets that could also occur in angiosarcoma.

**Methods:**

Splenic hemangiosarcomas are common in dogs, which allowed us to collect a cohort of archived matched tumor and normal tissue samples suitable for whole exome sequencing. Mapping of the reads to the latest canine reference genome (Canfam3) demonstrated that >99% of the targeted exomal regions were covered, with >80% at 20X coverage and >90% at 10X coverage.

**Results and conclusions:**

Sequence analysis of 20 samples identified somatic mutations in *PIK3CA*, *TP53*, *PTEN*, and *PLCG1*, all of which correspond to well-known tumor drivers in human cancer, in more than half of the cases. In one case, we identified a mutation in *PLCG1* identical to a mutation observed previously in this gene in human visceral AS. Activating *PIK3CA* mutations present novel therapeutic targets, and clinical trials of targeted inhibitors are underway in human cancers. Our results lay a foundation for similar clinical trials in canine HSA, enabling a precision medicine approach to this disease.

## Introduction

Angiosarcomas (AS) are rare malignant tumors of the endothelium of blood vessels, comprising up to 1–2% of sarcomas in humans (Sarcoma Foundation of America, https://www.curesarcoma.org/patient-resources/sarcoma-subtypes/angiosarcoma/). AS is deadly, with a 5 year survival of less than 30%[1]. Disease pathogenesis is poorly understood and there are no effective therapies. AS presents in three clinical situations: post irradiation therapy, sun exposed skin in older individuals, and, less commonly, spontaneous disease in visceral organs. As a result of its rarity, little is known about potential driver mutations that could serve as therapeutic targets. There is some evidence that post irradiation AS have a different mutational spectrum than cutaneous AS [2]. Visceral AS have not been well studied at the molecular level. The rarity of AS has impeded efforts to identify driver mutations that could present therapeutic targets, and the prospects for accruing patients to clinical trials are poor. To address this problem, some investigators are using "crowdsourcing" to identify patients with this disease (@ASCaProject, Angiosarcoma Project, https://ascproject.org/home). A search of the Hospital of the University of Pennsylvania archives spanning several decades revealed only a few cases, and the quality of the DNA we extracted from archived paraffin blocks was too low to perform exome sequencing. We turned to comparative genomics, taking advantage of the observation that a histologically similar malignant tumor, hemangiosarcoma (HSA), is common in dogs, which would enable discovery (exome) sequencing of paired tumor-normal tissue samples.

Canine hemangiosarcoma (HSA) is a highly aggressive, fatal malignancy characterized by neoplastic cells of endothelial origin that form vascular structures histologically similar to human AS. HSA affects approximately 50,000 dogs per year in the United States[3,4]. As in humans, both visceral and cutaneous forms of the disease are recognized, but in dogs the visceral form, specifically splenic, is more common. Canine patients with visceral HSA usually present with hemoabdomen following rupture of the splenic mass. Despite emergency splenectomy and adjuvant chemotherapy, most dogs die within 6 months from pulmonary metastatic disease.

Based on the clinical and histopathological similarities to human visceral AS (S1 Fig), we hypothesized that dogs with HSA might serve as a tractable, spontaneous model system to identify potential therapeutic targets and to carry out clinical trials. If successful, this approach could provide key information applicable to human AS, and would also provide new tools to address this common, deadly disease in dogs. Our hypothesis that AS and HSA might share pathogenic features is supported by previous studies that have identified mutations and deletions in the same genes that are shared between HSA and AS, such as TP53 and PTEN [5–7]. More recently, genome-wide expression profiling identified three distinct tumor subtypes associated with angiogenesis (group 1), inflammation (group 2), and adipogenesis (group 3). These data suggest that a common HSA progenitor may differentiate into the three different tumor subtypes and raise the intriguing question of the dynamic tumor evolution in HSA[8]. Genome-wide array-based comparative genomic hybridization (aCGH) revealed a relatively low rate of copy number abnormalities with small amplitude in HSA[9]. These data suggest that, in contrast to some of the other sarcomas, the driving force behind HSA might not involve global, high-grade alterations at the chromosomal level. Rather, small insertions/deletions and single nucleotide variants may drive tumorigenesis. Genome-wide association studies of HSA identified two predisposing loci on chromosome 5 but failed to find coding changes[10]. Thus, neither the somatic driver mutations nor the nature of the pathways leading to oncogenesis in this common and deadly disease are known.

## Results

### Case collection and exome sequencing

**Fig 1:**
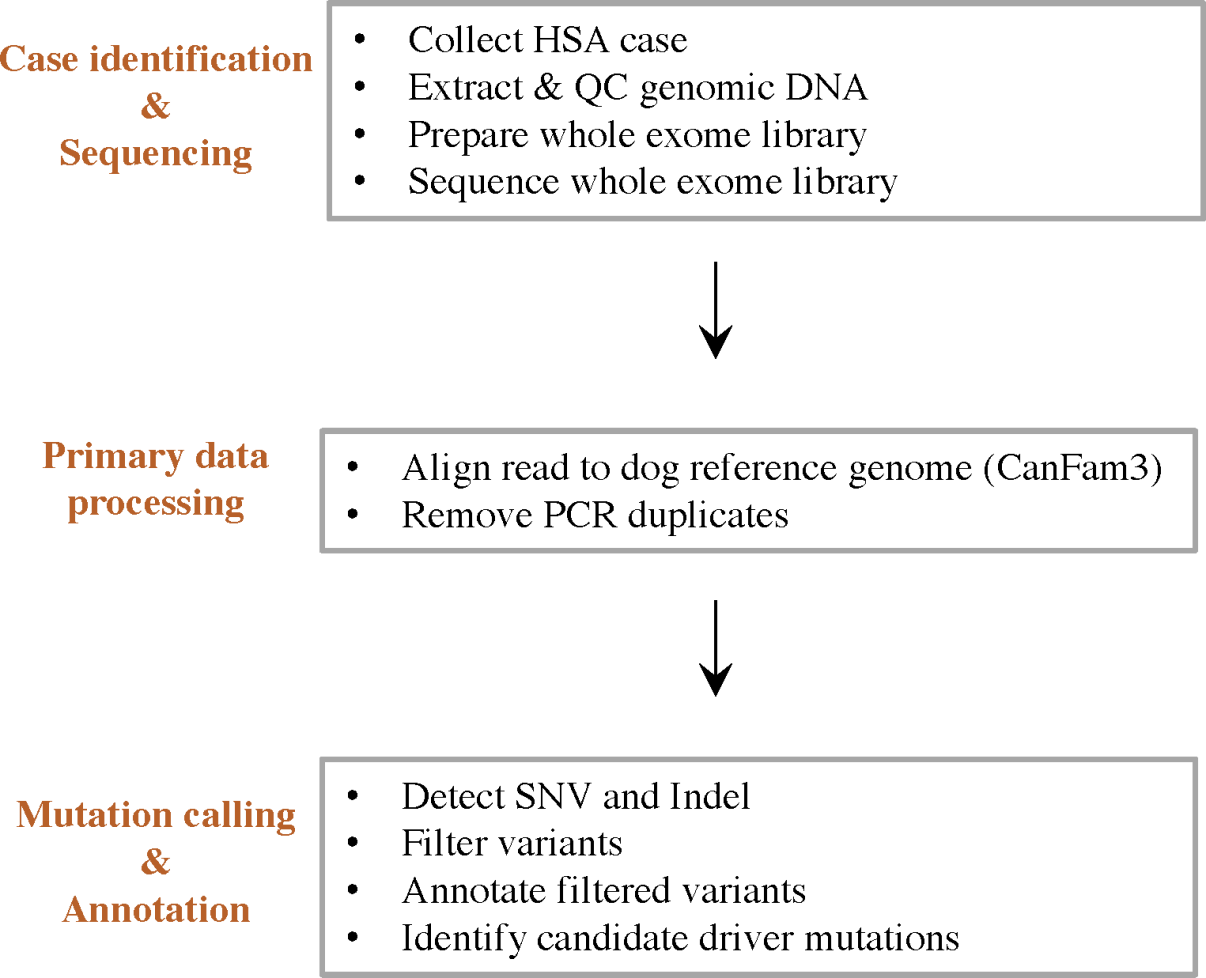
Whole exome sequencing workflow. **Case identification and sequencing**: Splenectomy samples (tumor and normal) were obtained from specimens derived from patients undergoing standard clinical care at our veterinary hospital. Genomic DNA were extracted and DNA quality was assessed via three QC steps. Exome libraries were prepared according to manufacturer’s protocol and sequenced on a Nextseq 500 at the Perelman School of Medicine sequencing core. **Primary data processing**: Reads were aligned to canine reference genome CanFam3 and PCR duplicates were removed. **Variant calling and annotation**: Sequence variants were detected, filtered and annotated by in-house pipeline. Candidate driver mutations were identified as summarized in Fig 2.

**Fig 2:**
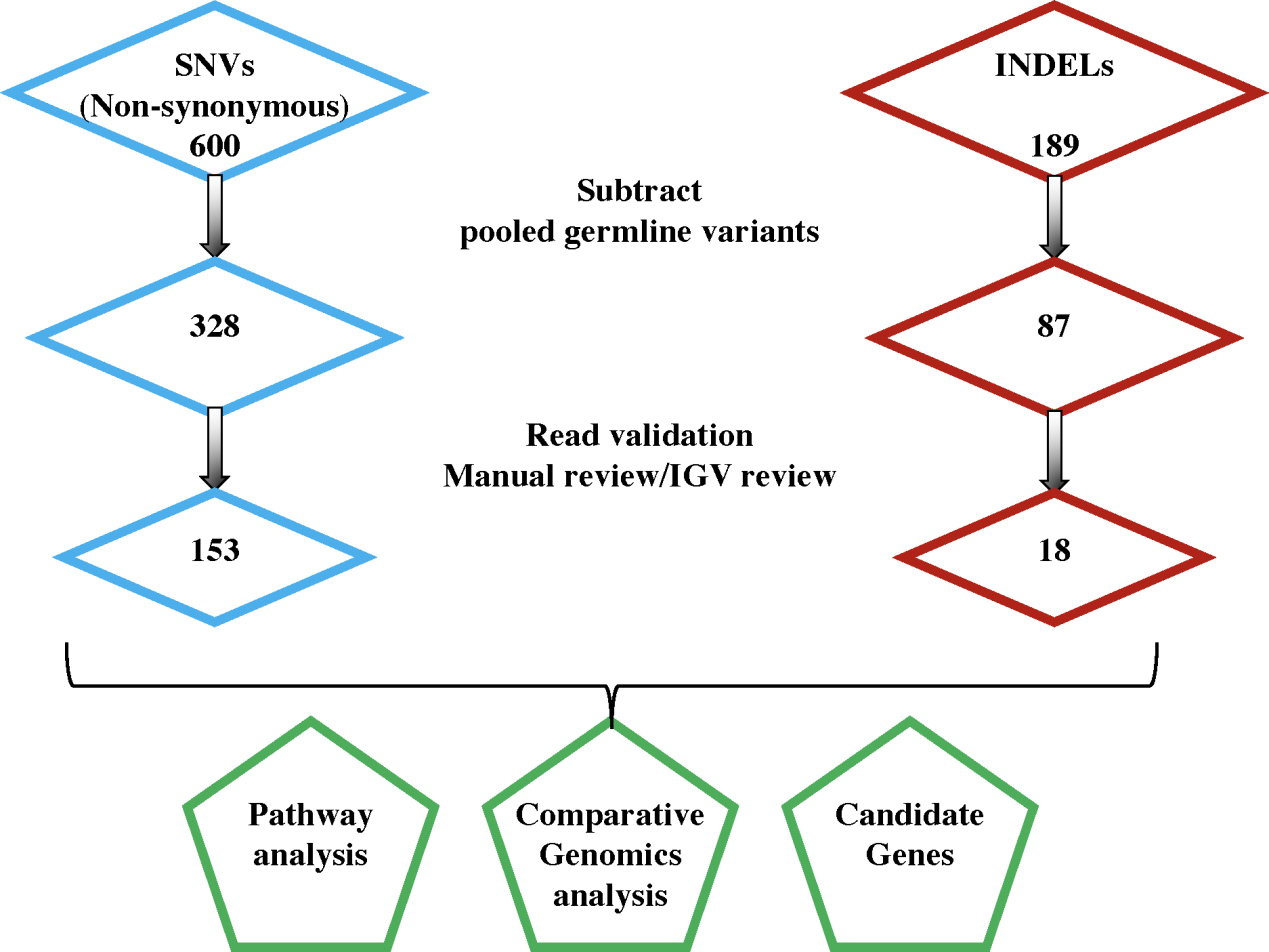
Decision tree for filtering and curation of variants. Single nucleotide variants (SNVs) and small insertion-deletion variants (indels) were filtered as shown, on the left and right sides of the Fig, respectively. The final filtered SNVs (167) and indels (32) were subjected to various annotation steps, such as pathway analysis, comparative genomics analysis and candidate gene comparison, as schematized at the bottom of the Figure.

We collected archived paired tumor and normal tissue samples from patients representing a variety of breeds, with a slight over-representation of Golden Retrievers, German Shepherd dogs and Labradors (S1 Table). We chose twenty-one cases for exome sequencing. DNA was extracted from microdissected tumor and matched normal formalin fixed, paraffin embedded (FFPE) tissue samples for whole exome sequencing (WES). We analyzed WES data following a bioinformatics workflow we customized for canine genomics, including mapping, mutation calling, and annotation (Fig 1; Materials and Methods). Mapping of the reads to the latest canine reference genome (Canfam3) demonstrated that >99% of the targeted exomal regions were covered, with >80% at 20X coverage and >90% at 10X coverage. Sequencing runs were designed to cover tumor at 2X the coverage of normal control DNA, as reflected by our data showing mean coverage of normal and tumor samples was 37X and 74X, respectively (S2 Fig). Sequencing data were filtered as described in Materials and Methods (Fig 2). One case (P2) was eliminated from the analysis because further review of stained slides from the dissected tumor revealed heavy contamination of the tumor sample by normal tissue. As expected, we observed no somatic mutations in that case.

**Fig 3:**
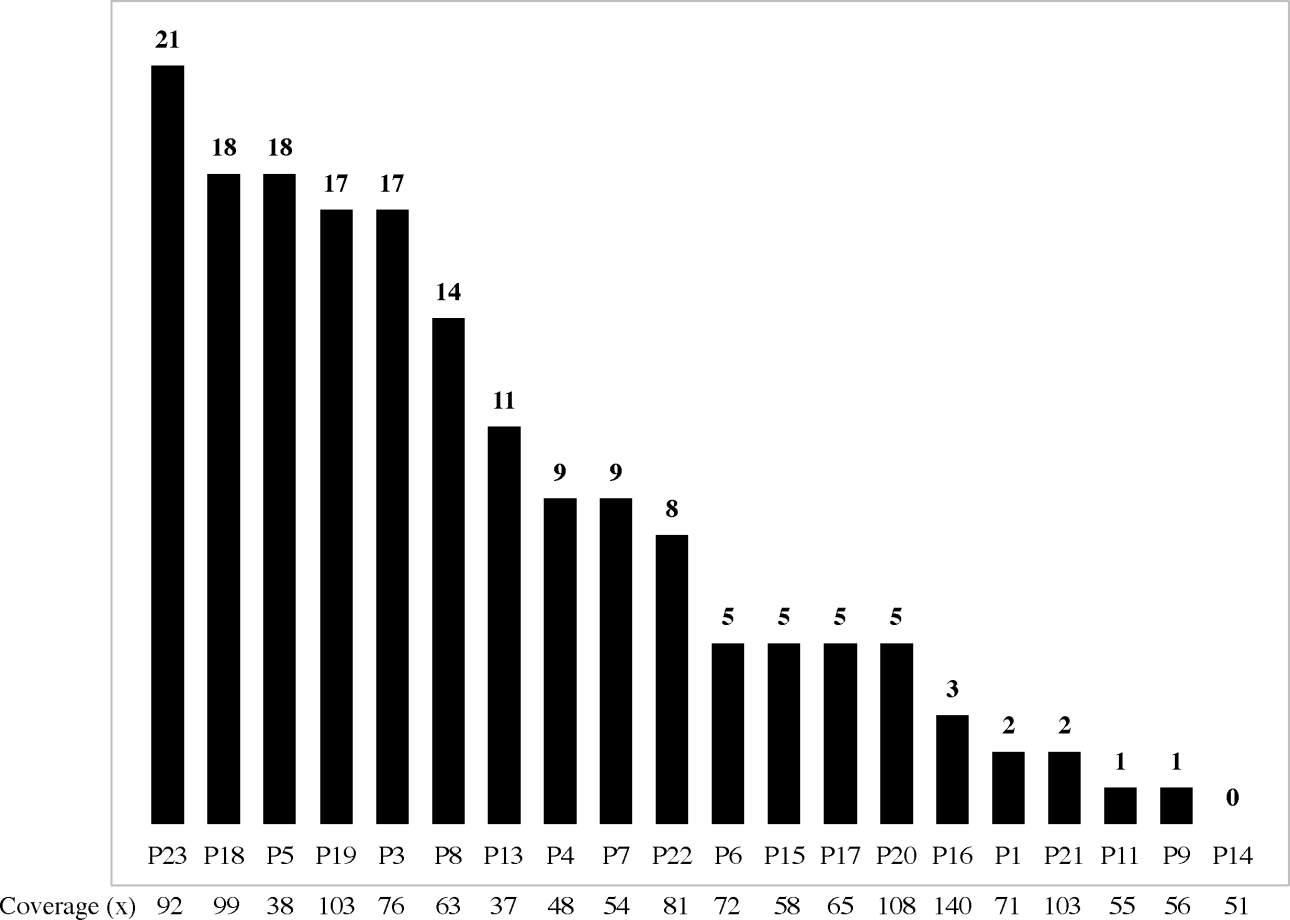
Number of mutations in each tumor. Nonsynonymous somatic mutation loads in canine HSA exomes: the numbers of somatic nonsynonymous mutations is displayed for each case of 20 HSA cases, ranked from left to right by the total number of mutations. Sequence coverage is listed under each case. Note that the number of mutations per case is not correlated with sequence coverage.

From these 20 cases, we identified 171 nonsynonymous somatic mutations: 153 single nucleotide variants (SNVs) and 18 insertions or deletions (indels). On average, we detected 8.6 missense somatic mutations per case (range, 0–21; Fig 3), and 9 or fewer missense mutations in more than half of our cases (Fig 3). Totaling the number of both nonsynonymous and synonymous mutations, the calculated HSA mutation burden is estimated to be 0.1–2.1 mutations per megabase, which falls on the low end of the mutation burden observed in human tumors (ranging 0.001–400 mutations per megabase), and is comparable to human tumors with low mutational burdens such as ovarian cancer and sarcomas [11,12].

### Mutations corresponding to drivers of human cancer occur commonly in HSA

The relatively low mutation burden simplified our search for candidate driver mutations. We examined somatic mutations in depth using three approaches: pathway analysis as described in Materials and Methods (Fig 2), comparative genomics (comparing to well-curated human data), and a candidate gene analysis in which we compared mutations with an in-house curated cancer gene list including COSMIC consensus cancer genes, genes with previously described mutations in human AS and canine HSA, as well as genes residing in pathways that are affected in vascular cancers[2,13,14].

**Fig 4:**
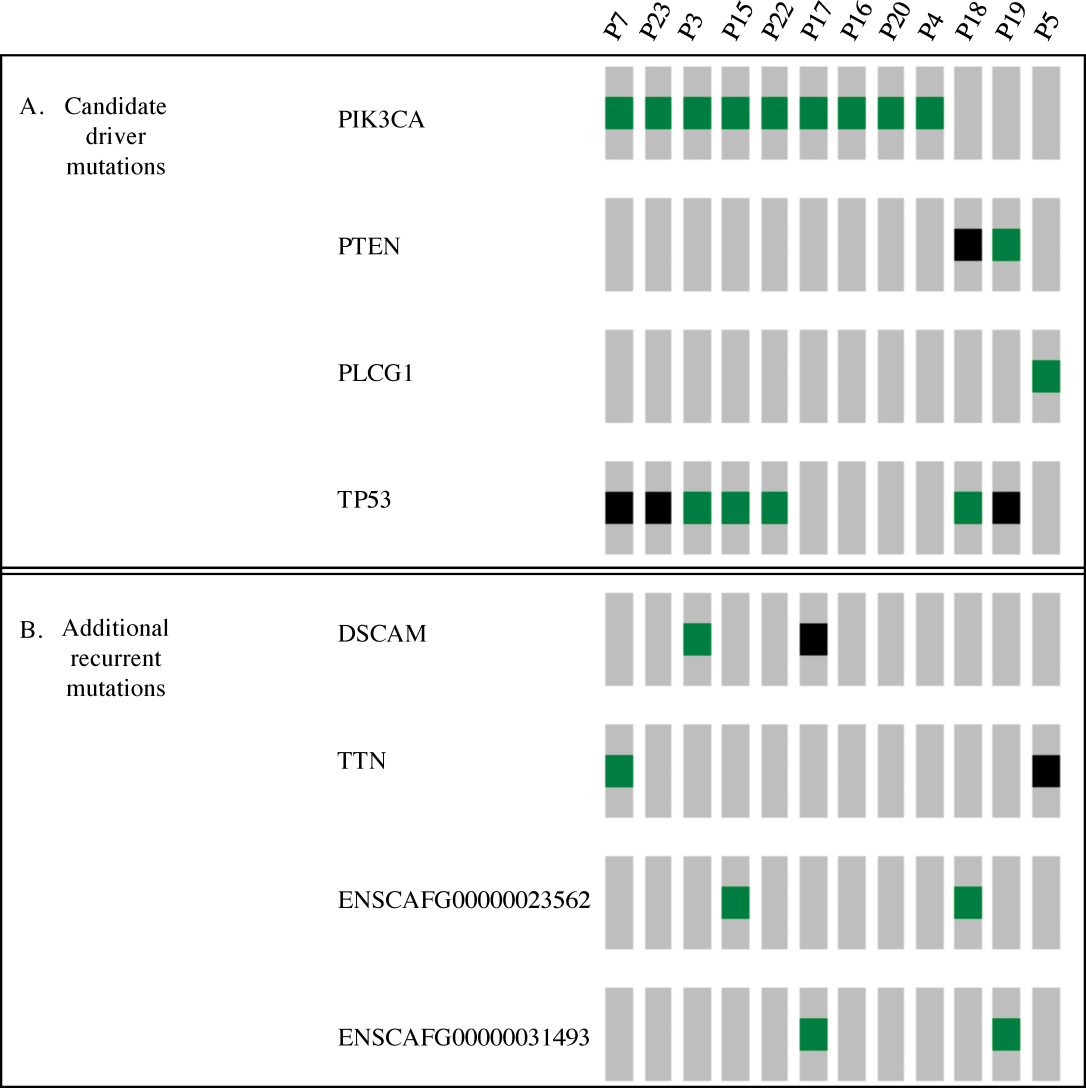
Candidate driver mutations and recurrent mutations. Each row represents a mutated gene and each column represents an individual tumor. Shown in green blocks are non-synonymous, non truncating variants which could represent gain-of-function mutations. Shown in black are predicted inactivating mutations, including truncating mutations, essential splice-site variants, and nonsense mutations. A: Candidate driver mutations, B: additional recurrent mutations.

Most informative was the cross-species comparative genomics approach, which allowed us to annotate canine mutations using human cancer-associated phenotypic data such as COSMIC, ClinVar, and HGMD (after converting to human amino acid coordinates as described in Materials and Methods). In over half of the cases (12/20), we identified somatic mutations predicted to have significant functional consequences in 4 genes previously established as human cancer drivers. We found *PIK3CA* mutations in 9 cases (45%), *TP53* mutations in 7 cases (35%), *PTEN* mutations in 2 cases (10%), and a *PLCG1* mutation in one case (5%), Fig 4A.

We next took a complementary approach to identify candidate driver mutations, which was to identify all recurrently mutated genes in our cohort, based on the hypothesis that some recurrently mutated genes might play a role in pathogenesis (Fig 4A and 4B). This analysis re-identified our strong driver candidates (*PIK3CA* mutations in 9 cases; *TP53* mutations in 7 cases; *PTEN* mutations in 2 cases). We also identified additional recurrently mutated genes: two mutations in two uncharacterized genes We also identified additional recurrently mutated genes: two mutations in two uncharacterized genes (ENSCAFG00000023562 and ENSCAFG00000031493), two mutations in *TTN* (likely passenger mutations [15], and two mutations in *DSCAM*. *DSCAM*, a large protein (1776 aa), is a negative regulator of cell adhesion and plays a role in neuronal self-avoidance, acting in central and peripheral nervous system development. Germline mutations in *DSCAM* have been reported in Down's syndrome and in congenital cardiac malformations [16,17]. We identified a *DSCAM* mutation not previously reported in cancer (Gly1679Ser; human: Gly1915Ser) and an Arg1562* mutation, whose human homologue, Arg1798*, has been identified in esophageal cancer [18]. Further investigation will be required to determine whether these mutations contribute to pathogenesis.

### Mutations in the PIK3CA pathway are likely drivers of HSA

**Fig 5:**
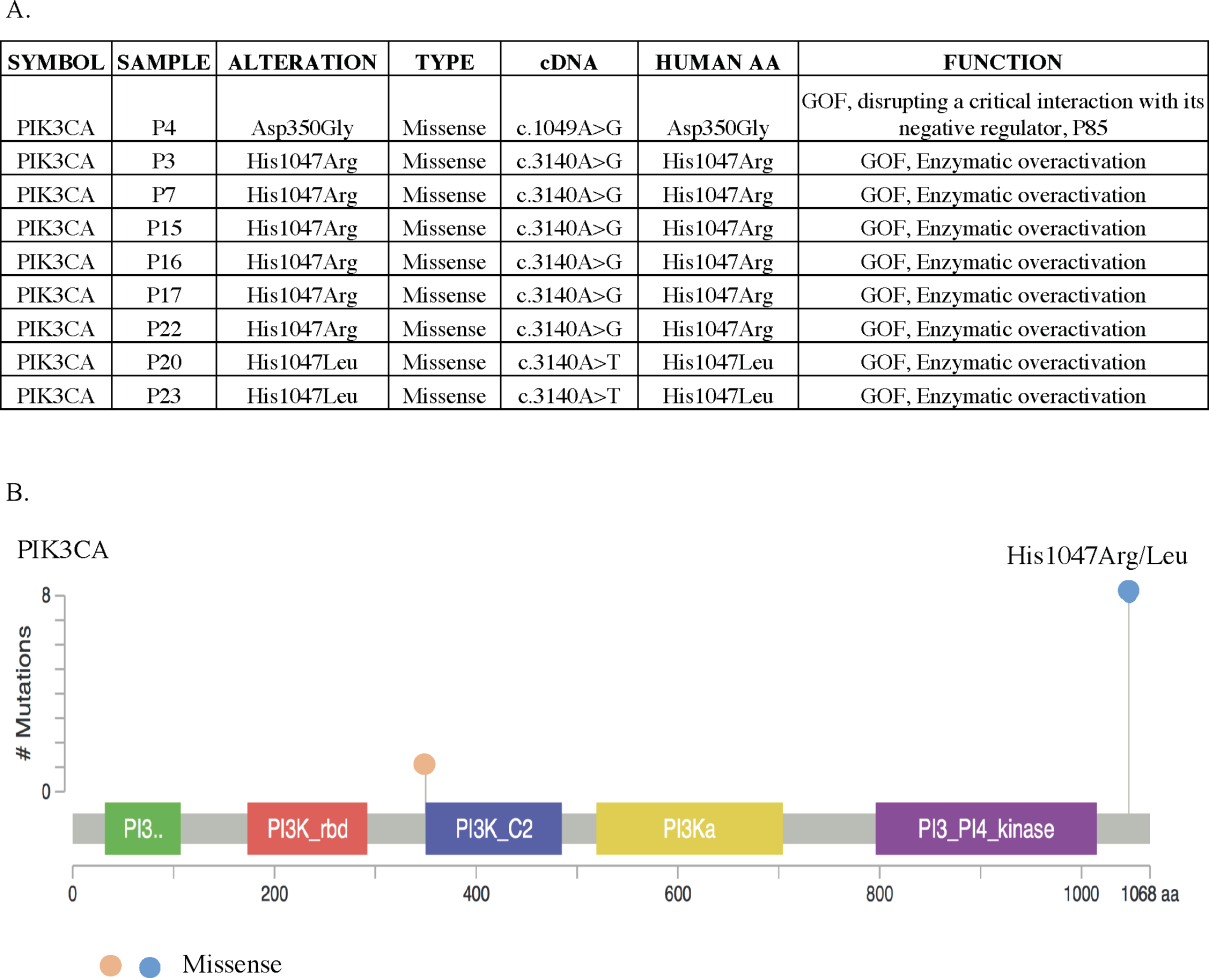
Distribution of alterations in the *PIK3CA* gene. (A) The table shows the distribution of *PIK3CA* mutations in our cohort of canine HSA cases, the corresponding human amino acid change, and the functional impacts of the mutants based on previous human studies. "GOF" denotes gain-of-function. (B) Schematic diagram of homologous mutations in human PIK3CA. The x axis represents amino acid positions and domain structure of human PIK3CA, and the y axis represents the number of times each mutation observed in our cohort.

The most frequent potential driver mutations affected the *PIK3CA* gene (9 cases; 45%). The human and canine PIK3CA genes share 99.8% sequence identity, and in 8 of our 9 PIK3CA mutant cases, the missense mutation affected amino acid 1047, the same position most commonly mutated in human cancers [19,20]. Indeed, six cases bear an H1047R substitution, a well-established driver mutation in human cancers (Fig 5). Two other cases have another nonconservative substitution at the same position (H1047L). In humans, H1047R is found in at least 4% of all cancers (COSMIC, http://cancer.sanger.ac.uk/cosmic), and accounts for almost 40% of all *PIK3CA* coding mutations (COSMIC, http://cancer.sanger.ac.uk/cosmic). The mutant protein shows increased enzymatic activity and activates downstream signaling, resulting in oncogenic transformation *in vitro* and tumorigenesis *in vivo* [19–22]. H1047L is also observed in human cancers, and both H1047R and H1047L are associated with a poor prognosis in adenocarcinoma of the lung [23]. Furthermore, clinical trials show that these pulmonary adenocarcinomas respond to *PIK3CA* inhibitors [24]. These data strongly suggest that the most prevalent mutations in *PIK3CA* we observed, H1047R and H1047I, function as cancer drivers in HSA.

In one case, we identified a *PIK3CA* D350G mutation, which has been identified in human breast, endometrial, pancreatic, and colorectal carcinomas [25,26]. Structural modeling of the human protein predicts that D350G disrupts interactions with PI3K signaling pathway inhibitors, and is therefore a gain-of-function mutation [27], suggesting that this mutation could drive canine HSA. Another indicator that the *PIK3CA* mutations we identified drive the development of HSA is that 7 of the 9 cases bearing *PIK3CA* mutations have fewer than 10 additional mutations (apart from those found in *PIK3CA* and *TP53*), and these are not obvious cancer drivers. Indeed, in one case (P16) bearing a presumed *PIK3CA* activating mutation (H1047R), only two additional mutations were identified, both in uncharacterized genes.

Our hypothesis that activation of the *PIK3CA* drives the development of HSA is further supported by the presence of other mutations affecting the PI3K signaling pathway in our cohort. *PTEN*, a negative regulator of the PIK3CA pathway, is mutated in two cases that conspicuously lack *PIK3CA* mutations. One *PTEN* mutation, shown in black in Fig 4A, is a frameshift mutation that encodes a truncated protein (Thr56fs). This heterozygous loss-of-function mutation is likely to have functional consequences, given that haploinsufficiency of the *PTEN* tumor suppressor gene promotes prostate cancer progression [28]. The second case, shown in green in Fig 4A, bears a missense mutation (Ile79Thr), affecting amino acid 79, which corresponds to amino acid 101 in the human protein. Although functional data are lacking, Ile101Thr is predicted to be highly pathogenic by FATHMM. In humans, Ile101 is mutated in several human tumors, primarily gliomas [29], suggesting that the canine PTEN Ile79Thr mutation may play a role in the pathogenesis of HSA.

**Fig 6:**
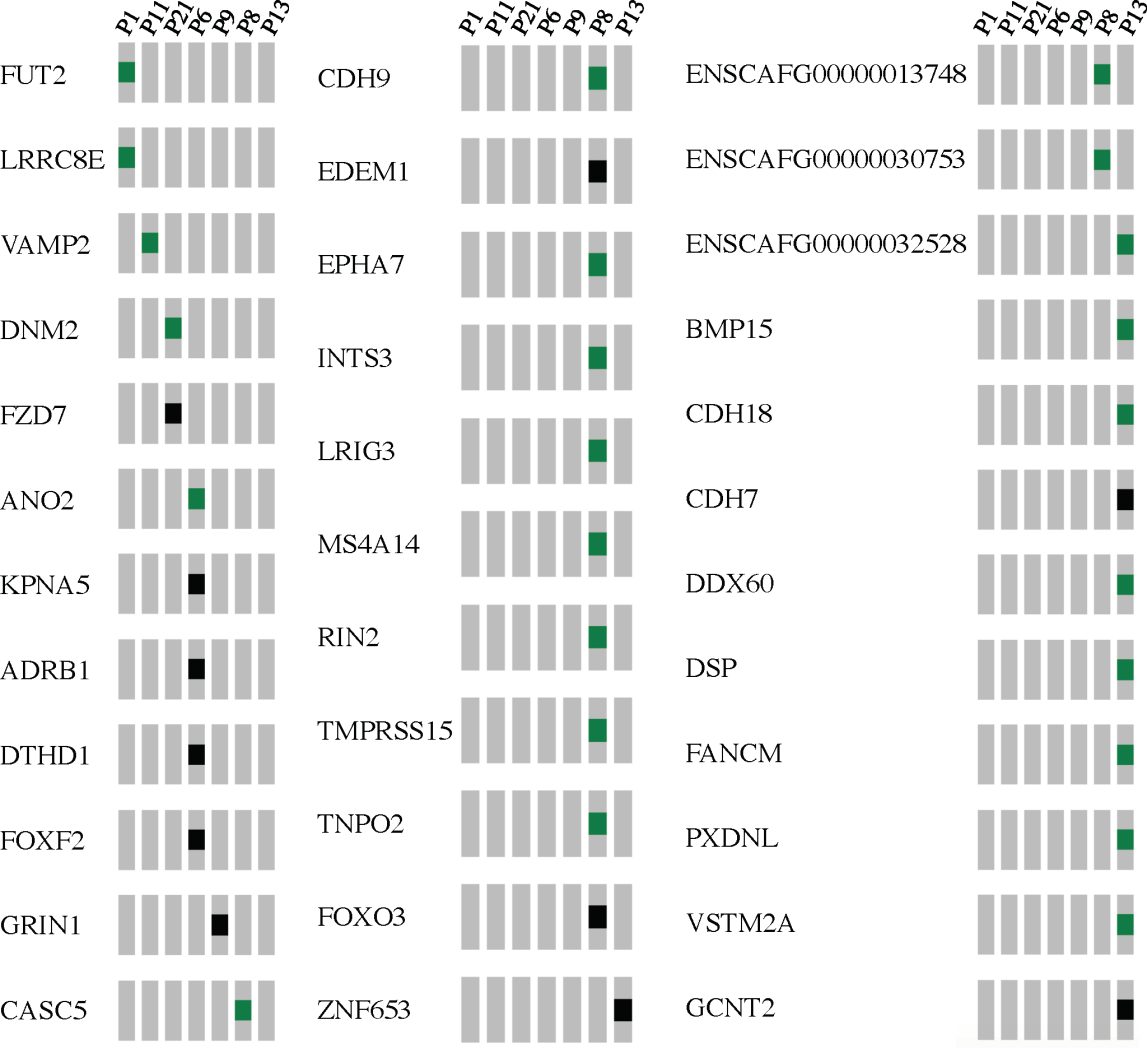
Summary of somatic mutations in HSA cases lacking strong driver mutation candidates. Green blocks denote missense mutations. Shown in black are predicted loss-of-function mutations (essential splice-site variants, nonsense mutations, and frameshift indels).

In another case, we observed a truncating mutation in *FoxO3* (Glu93fs), which appears to be a loss of function (P8; Fig 6). This case lacks *PIK3CA* and *PTEN* mutations. Previous work has shown, however, that activating PI3K signaling could negatively regulate Foxo3, leading to its cytoplasmic sequestration and degradation [30]. Thus, a loss of function mutation in *FoxO3* could activate downstream PI3K signaling, and may function in the same way as activation of *PIK3CA* or inactivation of *PTEN*.

### *TP53* is frequently mutated in HSA

**Fig 7:**
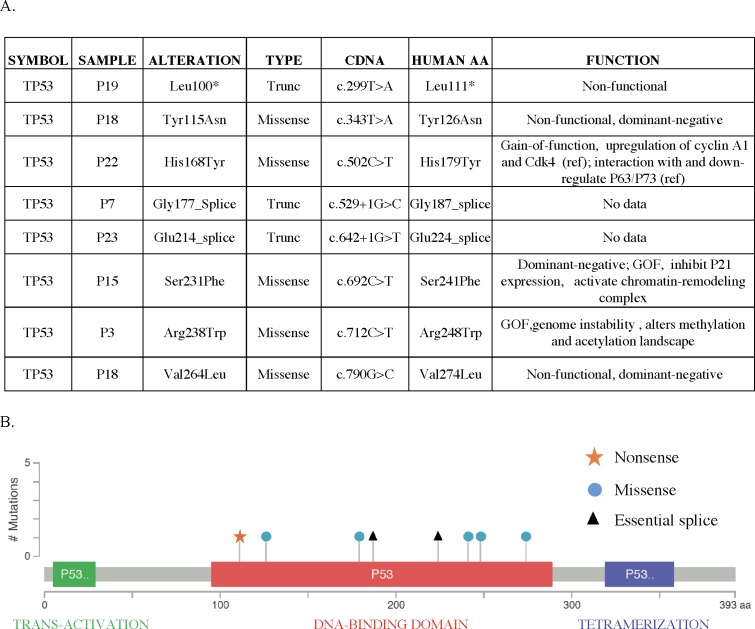
Distribution of alterations in the *TP53* gene. (A) Table shows the distribution of *TP53* mutations in our cohort, the mutation types, the corresponding human amino acid change, and the predicted functional impact of each mutation based on previous studies. (B) Schematic diagram of homologue mutations in human TP53. The x axis represents amino acid positions and domain structure of human TP53, and the y axis represents the number of times each mutation was identified in our cohort.

We identified *TP53* mutations in 7 cases (35%), making *TP53* the second most frequently mutated gene in our cohort (8 mutations in 7 tumors). All *TP53* mutations are predicted to have functional consequences, with truncating (1), missense (5), and essential splice site (2) mutations, all of which affect the protein's DNA binding domain (Fig 7). This is reminiscent of human cancer, in which more than 80% of *TP53* mutations localize to the DNA binding domain. Indeed, all of the canine *TP53* mutations we identified have been observed in human cancers, and some of them have been studied extensively. For clarity, we refer to amino acid positions in the human protein in the following discussion. In addition to the classic loss-of-function mutants (Leu111 nonsense mutation, Y126N), we also observed a dominant negative mutation (V274L) and identified several gain-of-function mutants. H179Y produces a growth advantage by upregulating Cyclin A1 and Cdk4 [31]. S241F acts as both a dominant negative and a gain-of-function mutant, inhibiting p21 expression and modifying chromatin remodeling patterns [32], and R248W induces genomic instability by inactivating ATM and also alters the epigenetic landscape [33,34]. Determining the mechanisms underlying the effects of these mutants requires further investigation.

#### A predicted activating mutation in *PLCG1* is homologous to a recurrent mutation in human AS

We observed a missense mutation in *PLCG1* (S273F) in one tumor. S273 is in the kinase domain of PLCG1 and is highly conserved among species. The corresponding human mutant, S345F, exhibits enzyme activation and increased downstream signaling, such as T cell receptor signaling and NF-kB signaling [35]. S345F drives tumorigenesis in human nodal peripheral T-cell lymphomas and cutaneous T-cell lymphomas [35,36], and this mutation was recently identified in human visceral AS [37]. These data suggest that canine *PLCG1* S273F contributes to the pathogenesis of HSA, and we speculate that this case might represent a subgroup of HSA with molecular similarity to human AS, especially the visceral form.

In conclusion, we identified mutations in 12 cases of canine HSA that, supported by abundant data from human cancers, likely play a role in oncogenesis in canine HSA. Apart from these strong driver candidate mutations, we identified a few mutations in genes potentially involved in cell growth, differentiation, or apoptosis (S2 and S3 Tables). However, none of the other mutations are found in human cancer databases or predicted to be involved in tumorigenesis. At this point, the data do not warrant implicating these mutations in HSA pathogenesis.

### HSA cases lacking obvious driver mutations

Eight cases lacked obvious candidate driver mutations. As noted above, analysis of recurrently mutated genes or comparative genomics failed to identify additional candidate driver mutations. All 36 mutated genes identified in these cases are schematized in Fig 6 and S2 Table. Our analysis failed to reveal candidates with sufficient evidence in the literature to support a clear role in pathogenesis of HSA. Clarification of the pathogenic drivers in these cases, which could involve structural variants or epigenetic changes not identified by exome sequence analysis, must await further investigation.

## Discussion

The recent development of canine exome capture reagents has opened the door for detailed profiling of canine cancer genomes. Our study represents the first use of this technology, as far as we are aware, to examine somatic genomic alterations in canine HSA. Sequencing a cohort of 20 HSA cases, we identified recurrent mutations in more than half of the cases whose human homologues are well-established cancer drivers, strongly suggesting that these candidates indeed drive canine HSA. Only one of our cases contained an apparent driver mutation (in PLCG1) which corresponds to mutations identified in human AS by targeted sequencing.

### Molecular pathways driving the pathogenesis of canine HSA

Our exome sequencing approach revealed that many cases of canine HSA harbor somatic point mutations that are likely to upregulate the PI3K pathway, which is well-established in human carcinogenesis. Our hypothesis that these mutations drive HSA pathogenesis is substantiated by previous work that demonstrated constitutive activation of the mTORC2/Akt/4E-BP1 pathway (by Western blot and immunohistochemistry) in newly derived canine HSA cell lines [38,39]. Indeed, viability of cell lines derived from HSA was significantly reduced by PI3K/AKT/mTOR inhibitors [40], and a PI3K inhibitor slows growth of primary cells derived from canine visceral, cutaneous, and cardiac HSA [41].

Pathogenic PIK3CA mutations also underlie human segmental hypertrophy syndromes, which commonly involve proliferative vascular malformations. For example, somatic, mosaic *PIK3CA* activating mutations are present in CLOVES (Congenital Lipomatous Overgrowth with Vascular, Epidermal, and Skeletal anomalies) Syndrome [42]. Analysis of 6 patients revealed the following *PIK3CA* mutations: p.His1047Arg, p.Glu542Lys, and p.Cys420Arg [42]. Activating PIK3CA mutations are also present in syndromes that now fall under the umbrella of *PIK3CA*-related overgrowth spectrum (PROS), in which vascular malformations are often a key feature [43,44]. Inhibiting the PI3K pathway reduces proliferation of primary cells from these patients in culture, linking PIK3CA activation to pathogenesis [43]. The presence of activating *PIK3CA* mutations in these syndromes and in HSA suggests that these disorders may share mechanistic similarities.

Although the most common candidate driver mutations in canine visceral HSA in our study affected the PI3K pathway, we also identified one case in which a *PLCG1* mutation is a likely driver candidate. This corresponds to mutations identified in a few cases of human visceral AS [37]. Additional work is required to determine whether *PLCG1* mutations are a common feature of canine HSA, and whether this potential subtype has a distinct biological phenotype. Finally, our analysis failed to identify obvious driver candidates in 40% of the cases. This may be due to insufficient sequence coverage (failing to detect low allele frequency events). Alternatively, this situation might reflect technical limitations of exome sequencing approach, which does not readily detect structural variants (gene fusions, deletions, gene amplification) that are commonly found in other sarcomas, but which are not suspected to play a large role in HSA [9]. Although further work is required to identify potential drivers in these cases, our data raise the intriguing possibility that the clinic-pathologic entity known as canine HSA may consist of multiple molecular subtypes, which may require different therapeutic approaches.

The high frequency mutations affecting the PI3K pathway in our cohort suggests the possibility that clinical trials in canine patients could be useful in determining whether inhibition of this pathway provides clinical benefit. Such trials are feasible: the disease is common (easy to fully enroll a multi-armed study), and rapidly fatal (allows expeditious determination of efficacy of experimental therapies).

### Is canine HSA a potential model of human visceral AS?

The *PLCG1* mutation (canine S273F, corresponding to human S345F) observed in our cohort was recently reported in human visceral AS [37] in a study of a large cohort of 120 well-characterized human AS from different anatomic sites, including several cases of visceral AS, studied by targeted hotspot sequencing. Furthermore, another activating *PLCG1* mutation, Arg707Gln, has been observed in human AS, primarily secondary and cutaneous forms [2,37,45]. We also observed mutations in *TP53* with likely functional consequences in canine HSA, which have also been observed in human visceral AS [46]. These data suggest that subgroups of canine HSA might serve as a natural model for human visceral AS.

We did not, however, identify some known genetic alterations found in human AS, such as *MYC* and *KLT4* amplifications and inactivating *PTPRB* mutations. Furthermore, we did not observe the high frequency of genetic events affecting the MAPK pathway suggested to play a central role in human AS [41]. Likewise, no *PIK3CA* mutations have been reported in human AS, either by NGS panel sequencing [37,45] or by PCR screening [47]. These discrepancies could reflect technical limitations of the various studies, but may also be due to differences in the biology of the various subtypes of human AS. For example, human AS is most commonly seen secondary to therapeutic irradiation (e.g., for breast cancer) or chronic sun exposure (cutaneous AS), both situations in which a higher mutational burden is expected.

Taken together, our results advance diagnosis and potential therapy of both canine HSA and potentially human AS in several ways. First, our analysis provides candidates for targeted therapy in canine HSA. Second, our results provide a basis for rational construction of "hotspot", panel-based NGS sequencing approaches to facilitate classification of HSA cases in clinical trials of targeted therapeutics. Third, our identification of likely driver mutations highly homologous to those found in humans in a subset of HSA cases could facilitate development of panel-based sequencing approaches to allow interrogation of human visceral AS cases from archival FFPE tissue, perhaps ultimately resulting in targeted therapies.

## Methods

### Canine HSA cases and whole exome sequencing

Forty-two HSA tumor and matched normal formalin fixed paraffin embedded (FFPE) tissue blocks were collected at PennVet between 2015 and 2016 as part of standard clinical care. Microscopic slides were reviewed and confirmed by a veterinary pathologist (ACD); tumor and normal tissue for dissection were identified. FFPE tissue cores were harvested from tumor and normal tissue blocks using a 3 mm punch tool. Genomic DNA (gDNA)) was extracted using GeneRead DNA FFPE Kit (Qiagen) and was quantified using a Qubit 2.0 Fluorometer. We then accessed the quality of extracted gDNA with a TapeStation 2200 system. We prepared libraries from 23 HSA cases whose gDNA was of sufficient quality and quantity. Whole exome libraries were prepared for tumor and matched normal samples using Canine All Exon kit (Agilent technologies). Paired-end sequencing (2X75) was performed on Illumina NextSeq500 platform in the Next Generation Sequencing Core at the Perelman School of Medicine (NGSC, ngsc.med.upenn.edu). Sequence data were acquired and processed per NGSC instructions followed by uploading to Illumina BaseSpace sequence hub for downstream analysis. Sequence reads were aligned to the latest canine reference genome, CanFam3. Reads with a mapping quality less than 20 were filtered out, unmapped reads and PCR-derived duplicated reads were also eliminated using Picard (version:1.139)

### Variant detection

Somatic variants, both single nucleotide variants (SNVs) and small insertions/deletions (indels), were identified using VarScan (version: 2.3.9) with standard settings. We used SnpEff (version: 4.2) to annotate the variants for primary gene and protein information, as well as to predict basic functional impacts.

To identify somatic variants that are robust and functionally important, we implemented the following filters: 1) variants at the intergenic, intragenic, intronic, UTRs regions, as well as synonymous variants; 2) variants referenced in Dog SNPs (Broad Institute) and DoGSD: the Dog Genome SNP Database (http://dogsd.big.ac.cn/), applying a minor allele frequency threshold of 0.05; 3) variants found in any normal cases in this study 4) variants with read depth fewer than 10X. Finally, resulted variants were subjected to manual read validation and review using Integrative Genomics Viewer (IGV) to eliminate artifacts from the sequencing workflow.

### Variant annotation

We developed a pipeline to convert the canine mutations to human orthologues. First, we retrieved sequences of the altered canine proteins as well as human homologous proteins from ensemble, then we used BLASTp with smith-waterman algorithm to map the homologous proteins, with these parameters applied to identify orthologues: 1) e-value 0.0001 as the threshold to identify significant matches; 2) Gene symbols match between canFam and hg19; 3) more than 70% identity rate for sequence similarity. Finally, the mutated positions in the canine samples were mapped to the corresponding positions in the human gene using a custom script. We then performed downstream analysis with the human orthologues.

#### Pathway analysis

We applied pathway analysis to all pass-filter variants in order to find biological pathways (co-functioning genes/mutations sets) that are over-represented in the WES data, using major web-based databases, such as KEGG, Panther pathways, DAVID Bioinformatics database, ConsensusPathDB, etc. In addition, we also manually curated the mutated genes by ranking keywords extracted from Gene Ontology (GO) terms and from publications. In general, we found that the top-ranked enriched pathways are consistent between these two approaches.

## Supporting information

S1 Table Clinical features of canine hemangiosarcoma patients. (PDF)

S2 Table Additional somatic mutations in cases that have strong driver mutation candidates (shown in Fig 4). Highlighted in different colors are groups of mutated genes that might be involved in HSA pathogenesis.
(PDF)

S3 Table Additional somatic mutations in cases lacking strong driver mutations (shown in Fig 4). Highlighted in different colors are groups of mutated genes that might be involved in HSA pathogenesis.
(PDF)

S1 Fig Morphologic and immunohistologic similarities between canine HSA and human AS. Canine Visceral (Splenic) HSA and Human Visceral (Retroperitoneal) AS. Neoplasms share similar histologic features and are composed of plump polygonal cells, which line vascular spaces to form blood-filled clefts and cavities. There is marked nuclear and cellular pleomorphism and mitotic activity in both neoplasms.
(TIFF)

S2 Fig Mean sequencing coverage of tumors and matched normal tissue. (TIFF)
