# Supplementary material for: Actionable mutations in canine hemangiosarcoma
Source: PLoS One. 2017 Nov 30;12(11):e0188667. doi: 10.1371/journal.pone.0188667 (PMC5708669; doi:10.1371/journal.pone.0188667)
Supplement: S1 Table — (PDF) [file pone.0188667.s001.pdf]

Supplementary table1:clinical features of canine HSA patients

Sample ID	Patient ID	Breed	Age	Sex	Organ	Sample type
P1	B15-15919	Sheperd Mix	13y	MC*	Spleen	FFPE
P2	B15-16308	Bichon Frise	12y	MC	Spleen	FFPE
P3	B15-16309	Nova Scotia Duck Tolling Retriever	11y	FS*	Spleen	FFPE
P4	B15-16524	Beagle Mix	9y	MC	Spleen	FFPE
P5	B15-16550	Rottweiler Mix	10y	MC	Spleen	FFPE
P6	B15-16695	Labrador	10y	MC	Spleen	FFPE
P7	B16-03139	Labrador	9y	MC	Spleen	FFPE
P8	B16-00876	Soft-coated Wheaten terrier	12y	MC	Spleen	FFPE
P9	B16-00977	Not specified	7y	FS	Spleen	FFPE
P11	B16-00108	Golden Retriever	10.5y	M*	Omentum	FFPE
P13	B16-02324	German Shepherd	8y	FS	Liver	FFPE
P14	B16-03194	Cocker Spaniel/Poodle	11.5y	M	Spleen	FFPE
P15	B16-03440	Golden Retriever	12y	MC	Spleen	FFPE
P16	B16-00631	Portugese Water Dog	7y	M	Spleen	FFPE
P17	B16-00557	Labrador	10.5y	MC	Spleen	FFPE
P18	B16-03860	Labrador	10.5y	MC	Spleen	FFPE
P19	B16-04530	Bichon Frise	12y	FS	Spleen	FFPE
P20	B16-04722	German Shepherd	10.5y	F*	Spleen	FFPE
P21	B16-01238	Labrador	8y	MC	Spleen	FFPE
P22	B16-01301	Golden Retriever	9y	MC	Spleen	FFPE
P23	B16-01172	Golden Retriever	11.5y	MC	Spleen	FFPE

MC: Male Castrated, FS: Female Spayed, M: Intact Male, F: Intact Female
