# Supplementary material for: Actionable mutations in canine hemangiosarcoma
Source: PLoS One. 2017 Nov 30;12(11):e0188667. doi: 10.1371/journal.pone.0188667 (PMC5708669; doi:10.1371/journal.pone.0188667)
Supplement: S2 Table — Highlighted in different colors are groups of mutated genes that might be involved in HSA pathogenesis. (PDF) [file pone.0188667.s002.pdf]

Supplementary table 2
Additional Mutations in cases with strong candidate driver mutation

Case	PIK3CA	TP53	PTEN	PLCG1	Additional Mutations
P23	+	+			AKIP1, ALAD, ANPEP, CBLC, CST6, ENSCAFG00000008063, FRK, HPDL, MSX2, PCDH7, SCN7A, SMCO2, TEK, TMEFF2, TMEM132B, UBQLN3, ZFP36L1, C19orf68, PTPN3
P7	+	+			ADAMTSL1, FRMPD4, GABRA3, GDF5, GRIK1, TTN, IDO1
P22	+	+			EGFLAM, ENSCAFG00000013598, ENSCAFG00000018611, GABRA1, HDC, TYRO3
P3	+	+			BEGAIN, CDKL4, DACT3, DRP2, DSCAM, ENSCAFG00000002455, ENSCAFG00000007236, ENSCAFG00000007873, ENSCAFG00000032241, KRT3, SEMA3E, TDRD6, TRIM77, CDK16, SRRM4
P15	+	+			ENSCAFG00000023562, COL24A1, SYCE1
P4	+				AFG3L2,ENSCAFG00000023121,ENSCAFG00000024414,ENSCAFG00000024414,MCF2,NRM,RNF34,SLC22A10
P17	+				B3GALT5,DSCAM,ENSCAFG00000031493,TFPI
P16	+				ENSCAFG00000030853, ENSCAFG00000032269
P20	+				ENSCAFG00000002855,ENSCAFG00000029964,ENSCAFG00000031329,CEP89
P19		+	+		AASDHPPT,APOB,C3orf38,ELP4,ENSCAFG00000024022,ENSCAFG00000031493,FAT3,MMP10,PMFBP1,SMPD2,TMPRSS11D,TRIP11,WDR47,WDR6,C15orf27
P18		+	+		ADCY2,CPD,DPH1,ENSCAFG00000019057,ENSCAFG00000023562,GIF,GLP1R,LAMC3,LRRC39,MEI4,PLB1,TGM6,TRIM22,USP21,VDAC2
P5				+	ATP10A,ATP6V0D2,CLEC4F,ENSCAFG00000007869,ENSCAFG00000028629,FRAS1,GRM5,HEG1,IREB2,KANSL1,KRT20,MYH7,SQSTM1,TRPV2,TTN,OTULIN

Red: cancer genes (NCG5.0 network of cancer genes, cosmic census genes)
Green: angiogenesis or PI3K signaling pathway
Blue: recurrent mutatation in human angiosarcoma
Pink: genes of potential relevance to tumorigenesis, such as those involved in proliferation, apoptosis, differentiation, etc.
