# Supplementary material for: Actionable mutations in canine hemangiosarcoma
Source: PLoS One. 2017 Nov 30;12(11):e0188667. doi: 10.1371/journal.pone.0188667 (PMC5708669; doi:10.1371/journal.pone.0188667)
Supplement: S3 Table — Highlighted in different colors are groups of mutated genes that might be involved in HSA pathogenesis. (PDF) [file pone.0188667.s003.pdf]

Supplementary Table 3
Mutations in cases w/o candidate driver mutation

Case	Number	Mutations
P1	2	FUT2,LRRC8E
P11	1	VAMP2
P21	2	DNM2,FZD7
P14	0	
P6	5	ANO2,KPNA5,ADRB1,DTHD1,FOXF2
P8	14	CASC5,CDH9,EDEM1,EPHA7,INTS3,LRIG3,MS4A14,RIN2,TMPRSS15,TNPO2,FOXO3,ENSCAFG00000013748,ENSCAFG00000030753
P9	1	GRIN1
P13	11	ENSCAFG00000032528,BMP15,CDH18,CDH7,DDX60,DSP,FANCM,PXDNL,VSTM2A,GCNT2,ZNF653

Red: cancer genes (NCG5.0 network of cancer genes, cosmic census genes)

Green: angiogenesis or PI3K signaling pathway

Pink: genes of potential relevance to tumorigenes, such as those involved in proliferation, apoptosis, differentiation, etc.
