# Supplementary figures and images for: Actionable mutations in canine hemangiosarcoma
Source: PLoS One. 2017 Nov 30;12(11):e0188667. doi: 10.1371/journal.pone.0188667 (PMC5708669; doi:10.1371/journal.pone.0188667)

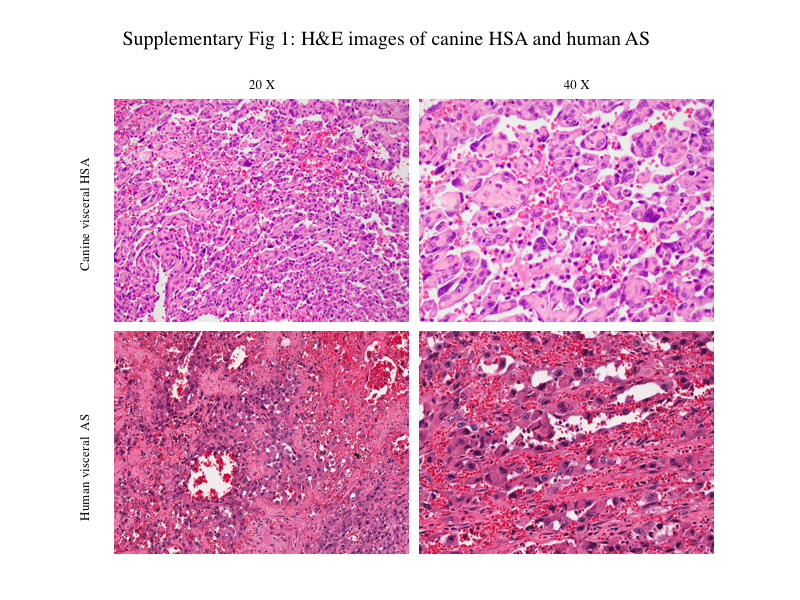

Supplement: S1 Fig — Canine Visceral (Splenic) HSA and Human Visceral (Retroperitoneal) AS. Neoplasms share similar histologic features and are composed of plump polygonal cells, which line vascular spaces to form blood-filled clefts and cavities. There is marked nuclear and cellular pleomorphism and mitotic activity in both neoplasms. (TIFF) [file pone.0188667.s004.tiff]

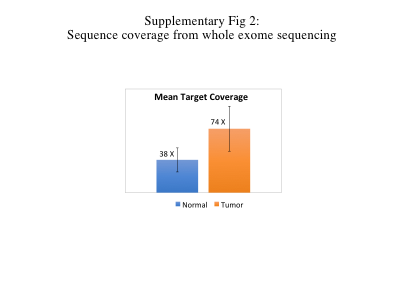

Supplement: S2 Fig — (TIFF) [file pone.0188667.s005.tiff]
